# Environmental and social determinants of cardiovascular risk in women with type 2 diabetes: a life-course perspective

**DOI:** 10.3389/fendo.2025.1667222

**Published:** 2025-10-08

**Authors:** Camilla Cocchi, Valentina Selleri, Giada Zanini, Federica Moscucci, Susanna Sciomer, Sabina Gallina, Milena Nasi, Giovambattista Desideri, Marcello Pinti, Claudio Borghi, Anna Vittoria Mattioli

**Affiliations:** ^1^ Department of Ageing, Orthopedics and Rheumatology, University “Cattolica del Sacro Cuore”, Rome, Italy; ^2^ National Institute for Cardiovascular Research (INRC), Bologna, Italy; ^3^ Department of Life Sciences, University of Modena and Reggio Emilia, Modena, Italy; ^4^ Azienda Ospedaliera Universitaria Policlinico Umberto I, Geriatric Unit, Rome, Italy; ^5^ Department of Clinical Medicine, Life, Health and Environmental Sciences, University of L’Aquila, L’Aquila, Italy; ^6^ “Sapienza” University of Rome, Rome, Italy; ^7^ Department of Neuroscience, Imaging and Clinical Sciences, “G. d’Annunzio” University of Chieti-Pescara, Chieti, Italy; ^8^ Department of Surgical, Medical and Dental Department of Morphological Sciences related to Transplant, Oncology and Regenerative Medicine, University of Modena and Reggio Emilia, Modena, Italy; ^9^ Department of Clinical and Internal Medicine, Anesthesiology and Cardiovascular Sciences, “Sapienza” University of Rome, Rome, Italy; ^10^ Department of Medical and Surgical Sciences Alma Mater Studiorum University-IRCCS AOU S. Orsola-Malpighi, Bologna, Italy; ^11^ Department of Quality of Life Sciences, Alma Mater Studiorum University, Bologna, Italy

**Keywords:** women, cardiovascular health, diabetes mellitus, socioeconomic determinants, environmental pollution, chronic stress, climate change

## Abstract

**Background:**

Cardiovascular disease (CVD) remains the leading cause of morbidity and mortality among women with type 2 diabetes (T2DM). The interplay between sex-specific biological factors, social determinants, and environmental exposures amplifies cardiometabolic risk across the female life course.

**Objectives:**

This manuscript explores how socioeconomic disparities, environmental pollution, chronic stress, food insecurity, and climate change synergistically increase the burden of T2DM and cardiovascular complications in women, and reviews potential preventive interventions including dietary strategies.

**Methods:**

A comprehensive narrative review was conducted, synthesizing current evidence on the exposome, social inequities, environmental insults, and evidence-based lifestyle interventions that contribute to or mitigate the development and progression of T2DM and CVD in women.

**Results:**

Lower socioeconomic status, limited education, housing instability, and inadequate access to healthcare and nutritious foods profoundly affect T2DM management and CVD prevention in women. Concurrently, exposure to air pollutants (PM_2.5_, NO_2_, O_3_), climate change-induced food insecurity, and heat-related stress further exacerbate insulin resistance, systemic inflammation, and vascular dysfunction. Life transitions such as gestational diabetes mellitus and menopause further magnify these risks. Current healthcare models insufficiently address these multilayered factors.

**Conclusion:**

Effective cardiovascular prevention in women with T2DM requires a life-course approach that integrates biological transitions with environmental and social determinants to deliver sex-sensitive, stage-specific strategies.

## 1 Introduction

**Figure 1:**
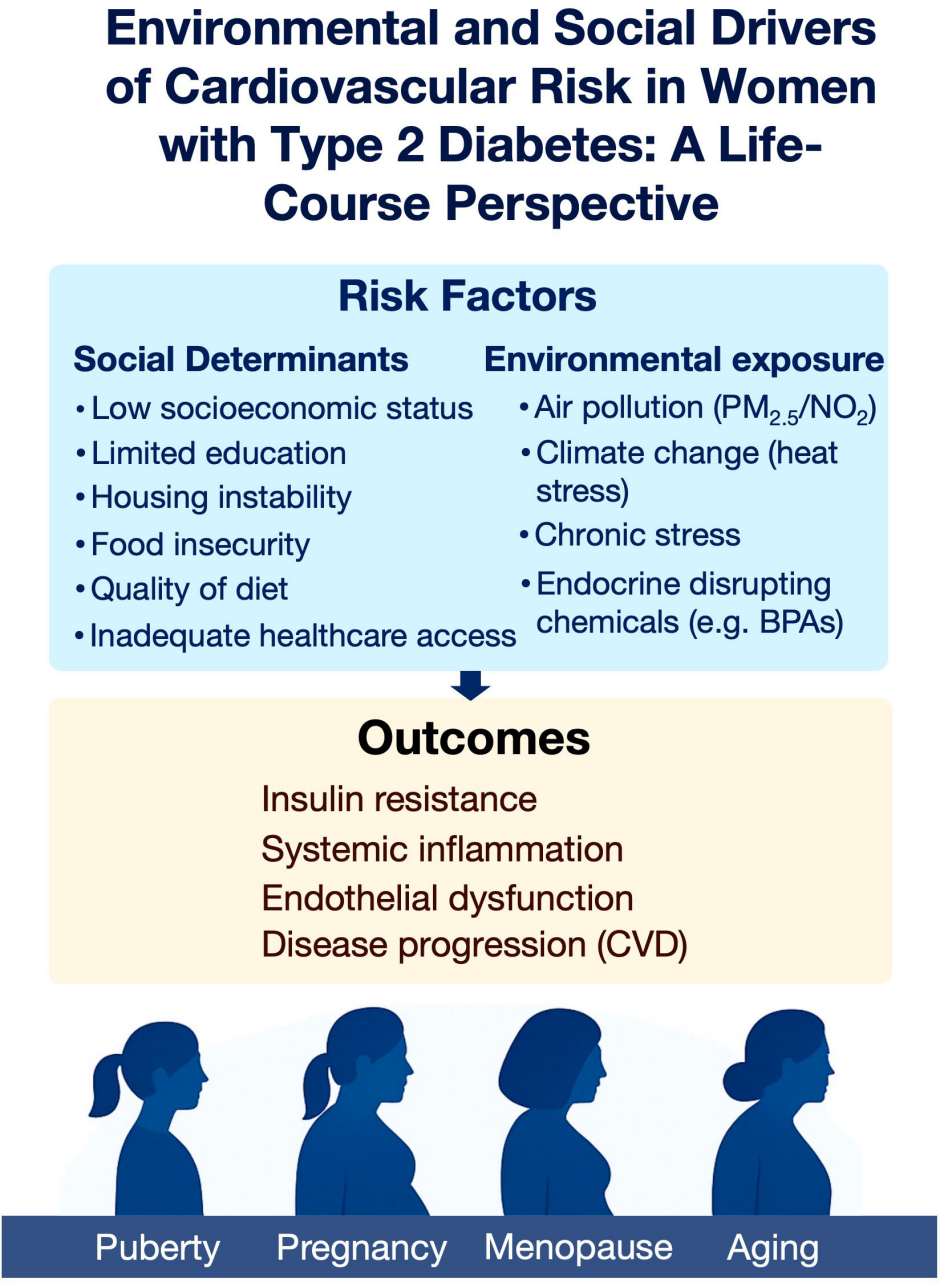
Environmental and social determinants of cardiovascular risk in women with type 2 diabetes across the life course. Social and environmental stressors converge to promote insulin resistance, systemic inflammation, endothelial dysfunction, and cardiovascular disease progression. These risks vary in impact across key life stages, including puberty, pregnancy, menopause, and aging.

The prevalence of type 2 diabetes mellitus (T2DM) is rising globally, with women increasingly affected during key life stages such as pregnancy, menopause, and aging (1–3). Among women with T2DM, cardiovascular disease (CVD) risk is markedly higher, due to a combination of metabolic dysfunction, hormonal shifts, and social/environmental vulnerabilities (4, 5). This review specifically focuses on women with T2DM, examining how environmental and social determinants interact with hormonal transitions (puberty, pregnancy, menopause, aging) to outline cardiovascular risk across the lifespan (**Figure 1**). Cardiovascular risk persists across the lifespan, underscoring the necessity for dynamic, stage-specific strategies that adapt to women’s evolving biological and social contexts (6).

## 2 Methods

This narrative review was informed by a structured search of PubMed, Embase, and Web of Science from January 2005 to March 2025. Search terms included combinations of “women,” “diabetes,” “cardiovascular disease,” “life course,” “hormonal transitions,” “puberty,” “pregnancy,” “menopause,” “aging,” “environmental exposures,” “social determinants,” “pollution,” and “climate change.” Inclusion criteria were peer-reviewed original research articles, systematic reviews, or meta-analyses focusing on cardiovascular risk in women with T2DM, published in English. Exclusion criteria were animal studies, conference abstracts, and papers without sex-disaggregated data. Reference lists of key articles were hand-searched to identify additional relevant publications. A simplified PRISMA flow diagram summarizing study selection is provided as **Supplementary Figure S1**.

To contextualize the strength of the evidence, we applied elements of the GRADE/WHO framework (7) to evaluate the certainty of evidence and risk of bias for each major exposure–outcome relationship. Factors considered included study design, risk of bias, consistency of results, and directness of evidence. The results of this assessment are presented in **Table 1**. In addition, to further interpret the strength and consistency of observed associations, we applied the Bradford-Hill criteria for causality, with particular attention to temporality, consistency, biological plausibility, and coherence with existing evidence (8).

**Table 1:** Certainty of evidence and risk of bias assessment for environmental exposures and T2DM related cardiovascular risk in women.

Exposure	Outcome	Main evidence base	Key limitations	Overall certainty (GRADE)
PM_2.5_ (air pollution)	T2DM incidence, CVD risk	Prospective cohorts; multiple meta-analyses (59, 79, 84, 85, 100)	Heterogeneity (I² not always reported), residual confounding, varying exposure assessment methods	Moderate – consistent association but some risk of bias
NO_2_/O_3_ (air pollutants)	T2DM incidence, CVD risk	Cohort and meta-analytic data (64, 73, 84)	Exposure misclassification, non-linear effects, less consistent for O_3_	Low–Moderate
Bisphenol A (BPA)	T2DM incidence, insulin resistance	Case-control, meta-analyses (80, 82, 86)	Heavy reliance on cross-sectional data, reverse causation possible, urinary measures vulnerable to misclassification	Low – suggestive but limited by study design
Endocrine-disrupting chemicals (EDCs, incl. phthalates)	T2DM risk, cardiometabolic outcomes	Observational studies, animal models (76, 87, 88)	Heterogeneity in exposure assessment, lack of longitudinal replication	Low
Noise pollution	Hypertension, metabolic dysregulation, CVD risk	Large European cohorts, ecological studies (64, 73)	Exposure misclassification, limited sex-stratified analyses	Moderate – consistent biological plausibility
Socioeconomic status/housing instability	T2DM complications, CVD risk	Systematic reviews, large cohorts (12, 42, 44, 49)	Residual confounding, variability in SES definitions	Moderate–High – consistent across contexts
Climate change (heat, food insecurity)	Glycemic instability, cardiometabolic outcomes	Ecological and cohort studies (65, 74, 75)	Indirect exposure assessment, emerging field	Low–Moderate

Although the literature search was conducted in a structured manner across multiple databases, a formal systematic review could not be undertaken. The available studies were highly heterogeneous with respect to design, populations, and reported outcomes, and many did not provide sex-disaggregated data. These factors limited the possibility of applying standardized quality assessment tools or conducting a meta-analysis. For these reasons, we chose to synthesize the evidence in the form of a narrative review, which allows for a broader and more integrative discussion of the topic.

## 3 Life stages, hormonal changes, and T2DM in women

Distinct life stages and hormonal transitions exert a profound influence on the pathogenesis and progression of T2DM and CVD in women (9). Critical physiological milestones, such as puberty, pregnancy, menopause, and aging, interact intricately with metabolic pathways, collectively heightening cardiovascular risk (1, 2, 9).

Puberty is characterized by a physiological increase in insulin resistance secondary to hormonal fluctuations, which may unmask subclinical metabolic dysfunction and predispose to early-onset T2DM (10). In adolescent females affected by obesity or polycystic ovary syndrome (PCOS), this risk is markedly exacerbated, yet often remains underdiagnosed and undertreated (11). Adolescents from socioeconomically disadvantaged backgrounds or those living in environments with limited access to healthy foods and safe recreational spaces face an amplified risk of early cardiometabolic dysfunction, highlighting the intersection of hormonal and environmental stressors during this stage. Targeted interventions during adolescence, including early screening for insulin resistance, promotion of healthy dietary habits, and structured physical activity programs, have been shown to yield enduring benefits in the preservation of cardiometabolic health (12, 13).

Pregnancy represents an additional critical window of vulnerability. Gestational diabetes mellitus (GDM), a condition affecting a substantial proportion of pregnancies, constitutes a powerful predictor of subsequent T2DM and CVD risk (14, 15). Women with a history of GDM exhibit a nearly sevenfold increased likelihood of developing T2DM within a decade postpartum and demonstrate elevated incidences of hypertension and metabolic syndrome (14, 16, 17). A cohort study from the Taiwan National Health Insurance Research Database (TNHIRD) analyzed a study population of 2,297,613 pregnant women. Women in the GDM cohort exhibited a significantly higher risk of developing T2DM, hypertension, and metabolic syndrome compared to the normal cohort, with hazard ratios of 7.07, 1.54, and 2.51, respectively. Similarly, the pregnancy-induced hypertension (PIH) cohort demonstrated an increased risk for these conditions, with hazard ratios of 3.41 for T2DM, 7.26 for hypertension, and 2.68 for metabolic syndrome. Notably, individuals with both GDM and PIH had the greatest risk, showing hazard ratios of 21.47 for postpartum T2DM, 8.02 for hypertension, and 5.04 for metabolic syndrome compared to the normal cohort (18). While observational data such as the Taiwan NHIRD cohort suggest possible interaction between GDM and PIH, most studies have not reported formal interaction terms or attributable proportions. Thus, evidence for true synergistic effects remains limited, and further analyses are needed to quantify these relationships. Women with a history of GDM are known to have a significantly elevated risk of developing T2DM (19, 20). A systematic review of 20 studies reported that individuals with prior GDM face an approximately sevenfold greater risk of T2DM compared to women without GDM (16). In a large population-based study, 18.9% of women with previous GDM developed T2DM within nine years after the index pregnancy, whereas only 2.0% of women without GDM were diagnosed with T2DM over the same period (16). Notably, the risk of developing T2DM was highest within the first nine months postpartum, during which 3.7% of women with prior GDM had already progressed to T2DM (21). Postpartum follow-up is often inadequate, representing a missed opportunity for early intervention (15). Structured screening, lifestyle counseling, and glucose monitoring post GDM are essential for long-term cardiovascular prevention (22, 23). Social determinants, including inadequate prenatal care access, food insecurity, and chronic psychosocial stress, may further increase the likelihood of adverse outcomes in women with GMD, thereby compounding future cardiovascular risk (22, 23).

Menopause represents a pivotal transition characterized by a decline in estrogen levels which worsens adiposity, insulin resistance, and dyslipidemia, thereby accelerating cardiovascular risk. Estrogen exerts protective effects on endothelial integrity and metabolic regulation; thus, its diminution is associated with increases in central adiposity, insulin resistance, and dyslipidemia (5, 6, 24). These metabolic disturbances exacerbate pre-existing diabetes-related cardiovascular risk, suggesting that the postmenopausal period can be a critical window for comprehensive risk reassessment and intensification of preventive and therapeutic strategies (25). Echocardiographic markers of hypertensive cardiac damage have been shown to provide valuable insights for such comprehensive assessment (26). The cardiovascular impact of menopause is magnified in women with lower SES or chronic exposure to stress and pollutants, which accelerate vascular aging and worsen postmenopausal metabolic dysfunction. Hormone replacement therapy (HRT), when initiated in selected populations with appropriate risk stratification, has been associated with a 20–30% reduction in new-onset T2DM. Its administration must be carefully individualized, taking into account the timing of initiation, overall cardiovascular risk profile, and the presence of comorbid conditions (27–30).

The aging process complicates T2DM management in women. In older patients, chronic hyperglycemia, oxidative stress, and inflammation accelerate frailty, sarcopenia, cognitive decline, and cardiovascular vulnerability (31–33). Multiple comorbidities, frequent hospitalizations, and heightened drug sensitivity further increase the risk of hypoglycemia, falls, fractures, and mortality (34). Cumulative life-course exposures, including socioeconomic disadvantage, air pollution, and social isolation, exert synergistic effects that worsen frailty, polypharmacy outcomes, and cardiovascular prognosis (6). Frailty is closely associated with both T2DM and hypertension through inflammatory, vascular, and metabolic pathways, with elevated cytokines such as IL-6 and TNF-α contributing to sarcopenia and diminished resilience (35, 36). Hyperglycemia promotes oxidative stress via excess reactive oxygen species and the accumulation of advanced glycation end products (AGEs), leading to cellular damage, tissue stiffening, impaired mobility, and accelerated ageing (35, 37). Endothelial dysfunction and reduced perfusion to muscle and brain further heighten risks of cardiovascular disease, stroke, and functional decline (36, 37). The burden of comorbidities such as cardiovascular and kidney disease compounds this trajectory (35, 37, 38).

To mitigate these outcomes, life stage–specific screening, personalized risk assessment, and culturally competent interventions are needed. Healthcare providers should adopt a proactive, multidisciplinary approach that integrates education, regular follow-up, and lifestyle modification aligned with hormonal and life-course transitions (13).

## 4 The exposome and diabetes-related cardiovascular risk in women

The following sections explore how socioeconomic, environmental, and lifestyle factors intersect with critical hormonal transitions across the female lifespan. Rather than acting uniformly, these determinants exert stage-specific effects, for example, socioeconomic status (SES) and stress strongly influence cardiometabolic trajectories during adolescence and pregnancy, while pollutant exposures and social isolation have cumulative impacts during menopause and aging. This life-course approach allows for a better understanding of CVD risk in women with T2DM.

In diabetic patients, the exposome concept helps identify cumulative environmental and social exposures that drive disease progression. For women with T2DM, pollution, poor nutrition, chronic stress, and socioeconomic disadvantages increase the burden of endothelial dysfunction, systemic inflammation, and atherosclerosis (13, 39).

Moreover, individual level socioeconomic status substantially modifies the vulnerability to environmental insults, with disadvantaged populations exhibiting heightened susceptibility. Collectively, these factors underscore the life-course influence of environmental and social determinants on the pathogenesis and progression of T2DM These risks are often compounded by insufficient healthcare access and sex-specific barriers to diagnosis and treatment.

### 4.1 Socioeconomic status, education, and T2DM

Women with T2DM who come from lower SES backgrounds encounter complex and interrelated challenges that elevate both the incidence and severity of cardiovascular complications (40–42). SES exerts a pervasive influence on multiple aspects of T2DM management and CVD prevention, including access to healthcare services, medication affordability, dietary quality, and opportunities for regular physical activity (43, 44). Individuals with lower SES are disproportionately affected by food insecurity, limited transportation to healthcare facilities, and restricted access to environments conducive to health promoting behaviors (45).

Nutritional quality in socioeconomically disadvantaged communities is frequently compromised due to the elevated cost and reduced availability of fresh fruits, vegetables, whole grains, and lean protein sources (12, 45, 46). Consequently, dietary patterns in these populations are often characterized by high intake of refined carbohydrates, added sugars, and saturated fats, dietary components that exacerbate insulin resistance and glycemic variability (46–48). This suboptimal nutritional profile contributes to impaired T2DM control and increases cardiovascular risk by promoting dyslipidemia and hypertension (12, 48).

Environmental factors such as housing instability, residential crowding, and unsafe neighborhoods reduce opportunities for physical activity, increase psychological stress, and are independently associated with cardiovascular morbidity (49, 50). These exposures contribute to sustained activation of neuroendocrine stress pathways, including the hypothalamic–pituitary–adrenal (HPA) axis, which promotes hypertension, insulin resistance, and vascular inflammation (51, 52). Chronic psychosocial stress, whether acute or cumulative (i.e., “allostatic load”), is now recognized as a key mediator linking social disadvantage to incident CVD events (52, 53). Incorporating housing insecurity into cardiovascular prevention frameworks is thus essential, particularly for diabetic women who already face heightened vulnerability. Chronic exposure to stress adversely affects hormonal homeostasis, impairing insulin sensitivity and elevating blood glucose levels, thereby intensifying cardiovascular risk (54, 55). These structural barriers are particularly detrimental for women, who frequently assume caregiving responsibilities and may prioritize the health needs of family members over their own (56, 57).

Beyond individual socioeconomic resources, the role of social support and community context is increasingly recognized as a determinant of cardiovascular health. Strong social networks buffer psychosocial stress, promote adherence to T2DM management, and enhance resilience against adverse events, whereas social isolation and weak community ties are linked with higher incidence of CVD and all-cause mortality (54, 55). For women with T2DM, the intersection of caregiving roles and limited community support may further exacerbate vulnerability to poor cardiovascular outcomes.

Educational attainment represents another critical determinant of health outcomes. Women with lower levels of education may have limited awareness of the symptoms and risks associated with T2DM and CVD and may face challenges in comprehending complex medical instructions, resulting in suboptimal adherence to treatment regimens and delays in seeking medical care (22, 43, 58, 59). In contrast, higher educational attainment is associated with enhanced problem solving skills, improved self-efficacy, and greater proficiency in navigating healthcare systems, all of which contribute to more favorable disease trajectories (58).

### 4.2 Environmental pollution and climate change

Environmental pollution and climate change are critical, yet frequently underrecognized, determinants in the development and progression of T2DM and its associated cardiovascular complications (59, 60). An expanding body of evidence links exposure to environmental pollutants, including fine particulate matter (PM_2.5_), nitrogen dioxide (NO_2_), and ozone (O_3_), to key pathophysiological processes such as insulin resistance, systemic inflammation, and oxidative stress, which underlie the onset of T2DM and atherosclerosis.

Air pollution contributes to endothelial dysfunction, inflammation, insulin resistance, and hypertension (61). Chronic exposure to PM_2.5_ and traffic-related pollutants is also linked to dyslipidemia, compounding risks for T2DM and cardiovascular disease (62). In addition to chemical pollutants, noise pollution, particularly from road traffic, rail, and air transport, has been independently associated with hypertension, metabolic dysregulation, and increased cardiovascular morbidity. Chronic nocturnal noise exposure disrupts circadian rhythms and sleep quality, contributing to sympathetic nervous system activation, insulin resistance, and endothelial dysfunction (59, 62). Women living in high-density urban areas with limited housing stability are especially vulnerable to these overlapping exposures.

The built environment influences opportunities for physical activity, diet, and stress regulation. Access to green spaces and walkable neighborhoods is associated with lower obesity rates, better glycemic control, and reduced cardiovascular risk. In contrast, poorly designed urban areas without safe recreational spaces or transport infrastructure promote sedentary lifestyles and widen health inequities (59, 61). For women with T2DM, supportive built environments can yield direct benefits (greater physical activity) and indirect ones (improved weight and glycemic control), thereby contributing to cardiometabolic health (63).

Environmental determinants such as the extent of green infrastructure (61), transportation modalities, and traffic density (62) modulate both local pollutant concentrations and individual patterns of physical activity. High levels of airborne pollutants may deter outdoor physical activity, while chronic exposure to environmental noise disrupts sleep architecture and adversely affects mental health, both of which are recognized contributors to cardiometabolic dysfunction.

Emerging studies also highlight the role of drinking water quality in cardiometabolic health. Contaminants such as arsenic, nitrates, and heavy metals have been linked to increased incidence of T2DM, endothelial dysfunction, and vascular disease (64). Ensuring equal access to clean, safe drinking water is therefore an essential component of cardiovascular prevention, particularly in socioeconomically disadvantaged communities.

Climate change further exacerbates these environmental threats through both direct and indirect mechanisms affecting metabolic health (65). Ultraviolet (UV) radiation exposure represents another underappreciated environmental factor. While excessive UV exposure contributes to oxidative stress, inflammation, and skin cancer risk, moderate exposure is essential for endogenous vitamin D synthesis, which exerts protective effects on cardiometabolic health (66). Women with T2DM may thus experience dual vulnerability to both vitamin D deficiency and environmental stressors linked with excessive UV exposure (67).

Rising global temperatures, altered precipitation patterns, and an increase in extreme weather events compromise agricultural productivity and disrupt food supply chains, diminishing the availability of fresh, nutrient-dense foods (68). These effects disproportionately impact low-income populations and women, who often face preexisting economic and geographic barriers to accessing healthy food options (66, 69, 70). Consequently, reliance on calorie dense, nutrient-poor processed foods increases, elevating the risk of obesity, T2DM, and cardiovascular disease.

In addition, climate change has been shown to alter the nutritional quality of staple crops (71). Elevated atmospheric carbon dioxide (CO_2_) concentrations reduce the levels of essential micronutrients such as zinc, iron, and protein in grains and legumes. Micronutrient deficiencies, particularly in individuals with T2DM, can impair insulin sensitivity, compromise immune function, and heighten susceptibility to infections and chronic inflammation (72, 73).

Heatwaves, a prominent manifestation of climate change, impose substantial cardiovascular strain, especially in individuals with T2DM. Heat stress may precipitate dehydration, electrolyte imbalances, and glycemic instability (65, 73). Furthermore, certain pharmacological agents commonly used in T2DM management, including diuretics and beta-adrenergic blockers, impair thermoregulatory responses, thereby increasing vulnerability to heat-related morbidity and mortality (72, 74). Beyond heat stress, climate change also influences cardiovascular risk through increased frequency of wildfires (leading to acute surges in PM_2.5_ exposure), flooding events (which compromise access to healthcare and medications), and shifting patterns of vector-borne diseases, all of which disproportionately impact vulnerable populations (65). These compounded exposures increase risk for women with T2DM, particularly in resource limited settings.

Women, particularly those who experience systemic inequalities in access to healthcare and disproportionate environmental burdens, are more adversely affected by compounded exposures to environmental and social stressors. Indeed, pregnant women with T2DM are especially vulnerable: environmental toxins and climate related stressors (e.g., heat, pollution) can impair both maternal and fetal health, increasing the risk of preeclampsia, preterm birth, and low birth weight (75–77).

Addressing these intertwined challenges necessitates systemic interventions. Policy initiatives aimed at reducing pollutant emissions, improving urban air quality, and promoting sustainable and resilient food systems are imperative (78). Public health strategies must prioritize environmental balance and the resilience of communities most susceptible to environmental degradation. Furthermore, healthcare providers should receive education regarding environmental risk factors and incorporate environmental health assessments into routine T2DM management.

### 4.3 Environmental exposures, endocrine disruptors, and T2DM risk: quantitative evidence and policy implications

Emerging scientific data supports a potential association between environmental exposures and the development of T2DM, particularly among women. A meta-analysis examining the impact of fine particulate matter (PM_2.5_) on metabolic health reported a pooled hazard ratio (HR) of approximately 1.11 per 10 µg/m³ increment (95% CI: 1.03–1.20), indicating a significant increase in the risk of T2DM with rising pollution exposure (79). These findings are supported by mechanistic studies that implicate PM_2.5_ in systemic inflammation, oxidative stress, and endothelial dysfunction, hallmarks of insulin resistance and cardiometabolic disruption (22, 23, 30).

To improve clinical interpretability, we translated relative risks into absolute risks. For example, the reported hazard ratio of 1.11 per 10 µg/m³ of PM_2.5_ corresponds to a meaningful difference in real-world settings. A woman with T2DM living in a moderately polluted European city (annual mean PM_2.5_ ≈ 20 µg/m³) would have an absolute 10-year risk of cardiovascular events that is approximately 2–3% higher than that of a woman in a rural area with PM_2.5_ levels around 10 µg/m³. This equates to a number-needed-to-harm of roughly 35–50 over a decade, depending on baseline cardiovascular risk.

Endocrine-disrupting chemicals (EDCs), such as bisphenol A (BPA), represent an additional and growing concern. A meta-analysis including over 40,000 individuals demonstrated that urinary BPA concentrations were associated with a 28% increased odds of T2DM (OR: 1.28; 95% CI: 1.14–1.44) (80). Notably, even short-term BPA exposure at the US Environmental Protection Agency’s “safe” dose of 50 µg/kg/day has been shown to impair insulin sensitivity within four days, as confirmed by euglycemic-hyperinsulinemic clamp studies in healthy adults (81).

Emerging data also point to a non-linear, U-shaped dose–response curve for BPA. A 2024 population study revealed increased metabolic and mortality risk at both low and high urinary BPA concentrations, with the greatest inflection observed at around 1.99 ng/mL (82). These findings challenge the notion of a clear “safe threshold” and underscore the potential for endocrine interference even at low doses.

Mechanistically, air pollutants such as PM_2.5_ and NO_2_ provoke systemic oxidative stress, disrupt vascular homeostasis, and activate inflammatory cascades, which impair insulin signaling and β-cell function (30, 51). In contrast, BPA acts primarily through estrogen and thyroid hormone receptor pathways, affecting pancreatic insulin secretion and promoting insulin resistance. Preclinical models have also demonstrated that BPA exposure in pregnancy can result in epigenetic modifications and β-cell abnormalities in offspring, suggesting a transgenerational diabetogenic effect (83).

These environmental threats are not equally distributed. Women from socioeconomically disadvantaged backgrounds and racial minorities are disproportionately exposed to both air pollution and EDCs (12, 30, 44). This inequity is compounded by barriers to healthcare access, healthy food, and safe physical environments. The intersection of environmental injustice with social determinants of health contributes to persistent disparities in T2DM incidence and cardiovascular outcomes (12, 48). **Table 1** provides a summary of the certainty of evidence and risk of bias for the environmental exposures most frequently reported in relation to T2DM and cardiovascular risk in women.

Furthermore, transgenerational evidence suggests that environmental exposures during critical life stages such as puberty or pregnancy can affect future generations through epigenetic inheritance. Studies in both humans and rodents have linked ancestral exposure to obesogenic or diabetogenic environments with metabolic dysfunction in descendants (83, 87). This finding reinforces the need for early-life and preconception interventions, especially in women of childbearing age.

While the literature on environmental pollutants and endocrine disruptors is extensive, other determinants such as housing instability and chronic stress also exert substantial and well-documented effects on cardiometabolic health.

Strengthening the evidence synthesis across these domains ensures a balanced representation of both established and emerging risk factors.

Targeted environmental policy reforms and public education campaigns will be essential to reduce the burden of T2DM in women and ensure equitable cardiometabolic health across generations.

Data are summarized in **Table 2**.

**Table 2:** Clinical and epidemiological studies on T2DM and environmental exposures, now including effect sizes, dose–response details, and mechanisms.

Study/cohort	Exposure	Outcome & population	Effect estimate	Dose-response/mechanism
Balti et al. (79)	PM_2.5_, NO_2_	T2DM incidence (Meta-analysis of five prospective cohort studies)	HR 1.11 per 10 µg/m³ PM_2.5_; HR 1.13 per 10 µg/m³ NO_2_	Adjusted for age, BMI, smoking. Inflammation & insulin resistance pathways implied
US Medicare older adults cohort (84)	PM_2.5_, NO_2_, O_3_	10M+ T2DM cases in 41M adults	HR 1.074 per 5 µg/m³ PM_2.5_; HR 1.055 per 5 ppb NO_2_; O_3_ not significant	Non-linear stronger effects at lower exposures (PM_2.5_ ≤ 8.2 µg/m³); PM→oxidative stress, endothelial dysfunction
Indian study (85)	PM_2.5_ annual ~30–100 µg/m³	12,000 adults	~22% higher T2DM risk per 10 µg/m³ PM_2.5_	Elevated fasting glucose; high urban pollution context
BPA trial (86)	Oral BPA 50 µg/kg/day × 4 days	40 healthy adults	↓ insulin sensitivity (clamp) vs placebo, p = 0.01	Short-term peripheral insulin resistance emerged without weight change
BPA–T2D meta-analysis (80)	Urinary/serum BPA	N ≈ 41,000 (13 cross-sectional, 2 case-control, 1 cohort)	OR 1.28 (95% CI 1.14–1.44); urine-only OR 1.20 (1.09–1.31)	Dose-response seen; mechanisms include β-cell and insulin signaling disruption
BPA study (82)	Urinary BPA	US adults, N ≈ 8,000	Non-linear U-shape for cancer mortality; lower BPA<1.99 ng/mL ↑ risk	Suggests low-dose endocrine disruption; possibly parallel for T2DM risk
Transgenerational EDC rodent study (87)	BPA (lower/higher doses) in pregnant dams	rodents	Germ cell (F2) and transgenerationally (F3) EDC-exposed females, but not F1, displayed delayed pubertal onset and altered folliculogenesis.	Dose-sex-gen-specific epigenetic effects
Disparities review (88)	EDC exposure (BPA, phthalates)	US by SES/race	Higher EDC levels correlate with T2DM disparities	Unequal exposures fuel health inequities

PM_2.5_, fine particulate matter; EDCs, Endocrine-disrupting chemicals; BPA, bisphenol A; NO_2_ nitrogen dioxide; O_3_, ozone.

### 4.4 The Mediterranean diet and prevention of metabolic and vascular complications in women with T2DM

Many studies support the role of the Mediterranean Diet (MedD) in mitigating the metabolic and cardiovascular consequences of T2DM. Several high-quality studies have demonstrated that adherence to the MedD reduces the risk of T2DM, enhances insulin sensitivity, and protects against diabetes-related vascular complications.

An umbrella review by Dinu et al. found that greater adherence to the MedD significantly reduced the risk of T2DM and several chronic diseases, reinforcing the importance of dietary interventions in cardiometabolic prevention (89). The PREDIMED-Reus study showed a lower risk of T2DM, particularly among female participants adhering to a MedD, emphasizing the need for sex-sensitive dietary research (90).

Multiple findings from the CORDIOPREV trial strengthen these conclusions. Boughanem et al. reported that women who adhered to a MedD showed a significant increase in insulin sensitivity and Disposition Index, especially in those with reduced neutrophil counts—a marker of improved inflammatory status (91). Another substudy of the CORDIOPREV study, authors demonstrated that improved diet quality through MedD adherence was associated with a lower risk of developing T2DM in patients with coronary heart disease (92). Jiménez-Torres et al. further confirmed the protective vascular effects of a long-term MedD enriched with extra-virgin olive oil, which was associated with reduced atherosclerosis progression (93). A recent meta-analytic review revealed that adherence to the MedD was correlated with a reduced incidence of diabetic complications, particularly nephropathy and retinopathy, underscoring the diet’s systemic protective effects (94). In the United States, the Keto-Med crossover trial by Gardner et al. compared a well-formulated ketogenic diet with a Mediterranean-Plus diet in patients with prediabetes or T2DM. Both interventions improved HbA1c, but LDL cholesterol improved more under the MedD, highlighting its cardioprotective metabolic profile (95).

These studies collectively suggest that the MedD as a clinically validated, sustainable strategy for improving metabolic control, preventing T2DM progression, and reducing the burden of vascular complications in women with T2DM. Although evidence generally supports the Mediterranean diet, it is important to acknowledge that the PREDIMED trial was retracted and subsequently republished with corrected analyses, and that the Women’s Health Initiative Dietary Modification trial did not demonstrate cardiovascular benefit. These controversies highlight that not all large-scale studies have shown consistent positive effects.

Ultimately, a holistic approach that recognizes environmental exposures as integral components of T2DM and cardiovascular disease prevention and management is essential for safeguarding the health of women living with T2DM in the context of a rapidly changing global environment. To further illustrate the dynamic interplay between life-course transitions and environmental or social determinants, we provide a synthesis linking hormonal and metabolic vulnerabilities with stage-specific risk factors and outcomes (**Table 3**). This integrated framework highlights how puberty, pregnancy, menopause, and aging each represent critical windows during which socioeconomic disadvantage, chronic stress, pollution, and other exposures exert differential effects on cardiometabolic health in women with T2DM. Such an approach underscores the necessity for prevention strategies that are both life-stage–sensitive and contextually tailored to women’s lived environments.

**Table 3:** Integration of risk factors and life-course stages in women with T2DM.

Life stage	Key hormonal/metabolic vulnerability	Exacerbating risk factors	Example outcomes
Puberty	Insulin resistance, PCOS	SES, built environment, food insecurity	Early obesity, T2DM onset
Pregnancy	GDM, PIH	Limited prenatal care, stress, poor diet	Future T2DM, HTN, CVD
Menopause	Estrogen decline, visceral fat	Pollution, stress, low SES	Dyslipidemia, vascular aging
Aging	Frailty, polypharmacy	Cumulative pollution, isolation	Cognitive decline, CVD events

To mitigate cardiovascular risk in diabetic women, comprehensive and multilevel interventions are essential. **Table 4** provides a structured overview of multilevel prevention strategies for women with T2DM, organized across social, environmental, lifestyle, life-stage, community, and health-system domains. By presenting specific examples alongside a qualitative grading of evidence strength, this framework illustrates both established interventions (e.g., Mediterranean diet, integrated care models) and emerging areas requiring further research (e.g., housing stability, endocrine disruptor regulation, climate resilience strategies). The table highlights how prevention must be tailored across the life course, from postpartum follow-up after GDM to frailty management in older age. While some strategies are supported by high-quality randomized evidence, others remain hypotheses for translational trials. Together, these strategies emphasize that effective cardiovascular prevention in women with T2DM will require coordinated action across clinical, community, and policy levels.

**Table 4:** Prevention strategies for women with T2DM.

Category	Strategy	Example interventions	Evidence strength
Social Determinants	Improve food access and affordability	Food-as-medicine programs, produce prescriptions, subsidies for healthy foods	Moderate (systematic reviews, quasi-experimental data) (22, 46, 48)
	Address housing and financial insecurity	Housing assistance, rental support, integrated social care in clinics	Low–Moderate (observational evidence) (49, 50)
	Enhance social support and stress resilience	Peer-support groups, workplace flexibility, community mental health programs	Low–Moderate (observational, emerging RCTs) (51, 52)
Environmental Pollution	Reduce exposure to air pollution and toxins	Clean-air legislation, low-emission transport zones, regulation of endocrine disruptors	Moderate (population-level evidence, natural experiments) (39, 40, 73)
	Mitigate climate-related health risks	Heat adaptation plans, urban greening, access to safe cooling centers	Low–Moderate (emerging but consistent epidemiology) (65, 75)
Nutrition & Lifestyle	Promote cardioprotective diets	Mediterranean diet, DASH diet interventions	High (multiple RCTs, meta-analyses) (89–91)
	Encourage safe physical activity	Urban walkability, green spaces, structured exercise programs	Moderate–High (reviews, emerging epidemiology) (25, 62)
Life-Stage Interventions	Early screening after GDM or PIH	Routine postpartum glucose testing, integrated CVD risk counseling	High (cohort studies, guideline recommendations) (16, 20, 22)
	Menopause transition care	Cardiometabolic screening, individualized HRT counseling	Moderate (observational, some RCTs) (28–30)
	Frailty and aging	Multidisciplinary geriatric- T2DM care, polypharmacy review	Moderate (systematic reviews) (31, 32, 38)
Community Initiatives	Build supportive environments	Safe recreation spaces, school-based health programs, culturally tailored interventions	Moderate (public health evaluations) (55, 63)
	Digital health engagement	Mobile coaching apps, remote glucose/CVD monitoring	Low–Moderate (implementation studies) (34)
Integrated Health Systems	Coordinate T2DM –CVD care	Joint endocrinology–cardiology clinics, team-based care models	High (meta-analyses of integrated care) (22)
	Incorporate environmental health in clinical care	Screening for pollution, housing, food insecurity in EHR with referral pathways	Emerging (conceptual, implementation pilots) (73)

This review has some limitations, particularly the reliance on observational studies and variability in reporting. The certainty of evidence varied across exposures: associations with PM_2.5_ and other air pollutants are supported by prospective cohorts and graded as moderate certainty, whereas evidence for BPA and other endocrine-disrupting chemicals remains low due to reliance on cross-sectional biomarker data, exposure misclassification, and vulnerability to reverse causation. Notably, substantial heterogeneity (I²) across air pollution studies limits the precision of pooled estimates. To interpret these findings, we applied the Bradford-Hill criteria for causality (96). While exposures such as PM_2.5_ meet several criteria including consistency, temporality, and biological plausibility, others such as BPA remain supported largely by associative data with limited experimental evidence and should not be overinterpreted as causal.

## 5 Future perspectives

Looking ahead, reducing cardiovascular risk in women with T2DM requires integrated strategies that bridge biology, environment, and social policy. Clean air and tobacco control legislation remain foundational, with well-documented benefits for population level cardiovascular outcomes. Expanding these approaches to include climate adaptation policies (e.g., urban greening, heat mitigation strategies, reduction of traffic related pollutants) is crucial, given the disproportionate impact of climate change on women’s cardiometabolic health. Nutrition focused policies such as “food-as-medicine” programs, produce prescriptions, and food bank partnerships have already shown benefits for diet quality, food security, and in some cases glycemic outcomes (97, 98). Embedding these programs within health systems and linking them to insurance reimbursement could enhance scalability and sustainability. At the clinical level, integrated care models, where endocrinology, cardiology, gynecology, and primary care coordinate, have been associated with reduced mortality and hospitalization in people with T2DM (99). Future innovations could incorporate digital health platforms (e.g., continuous glucose monitoring linked with cardiovascular monitoring, mobile health coaching) to personalize interventions across life stages. A promising frontier is the incorporation of environmental and social determinants into routine risk assessment. Screening tools for housing insecurity, food access, and environmental exposures could be embedded into electronic health records, enabling providers to identify high-risk women and connect them with tailored resources. Similarly, community level interventions, such as safe green spaces, walkable neighborhoods, and culturally tailored physical activity programs, can provide synergistic benefits.

Future research should prioritize sex-specific and life-course–specific trials that test whether targeting environmental exposures, social determinants, and hormonal transitions improves not only glycemic control but also cardiovascular outcomes and quality of life. Implementation science will be critical to scale up successful programs while ensuring equity. Ultimately, the next decade should focus on multisectoral interventions that simultaneously address medical, behavioral, environmental, and policy drivers of cardiometabolic risk. Such approaches hold the potential to transform care for women with T2DM from reactive disease management to proactive, prevention-oriented health promotion across the life course.

## 6 Conclusion

Women with T2DM face a complex interplay of cardiovascular risks shaped by biological transitions and by environmental and social determinants. This review adopts a life-course perspective, covering puberty, pregnancy, menopause, and aging, while highlighting cross-cutting factors such as socioeconomic status, chronic stress, pollution, and housing instability. As summarized in **Table 3**, some determinants (e.g., stress, food insecurity) exert their greatest impact in early life stages, whereas others (e.g., pollution, social isolation) accumulate and become more pronounced with aging. Recognizing these dynamic, stage-specific influences is essential for developing prevention and management strategies that are both sex-sensitive and life-stage specific. While pollutant exposure and dietary interventions such as the Mediterranean diet have been extensively studied, the effects of housing instability, chronic stress, and other social determinants remain comparatively underexplored. A comprehensive framework that integrates environmental hazards with modifiable lifestyle factors is needed to strengthen cardiovascular prevention in women with T2DM. Future translational research should prioritize evaluating how such systemic interventions can be embedded into clinical practice.

## Supplementary material

Supplementary Figure 1 Created in BioRender. Pinti, M. (2025) https://BioRender.com/1bnfxvg”. Footnote to supplemental material Flow chart. ”A formal systematic review was not feasible due to heterogeneity of study designs, outcomes, and lack of gender-disaggregated data; therefore, the evidence was synthesized narratively”.
