# Supplementary figures and images for: Environmental and social determinants of cardiovascular risk in women with type 2 diabetes: a life-course perspective
Source: Front Endocrinol (Lausanne). 2025 Oct 8;16:1667222. doi: 10.3389/fendo.2025.1667222 (PMC12540180; doi:10.3389/fendo.2025.1667222)

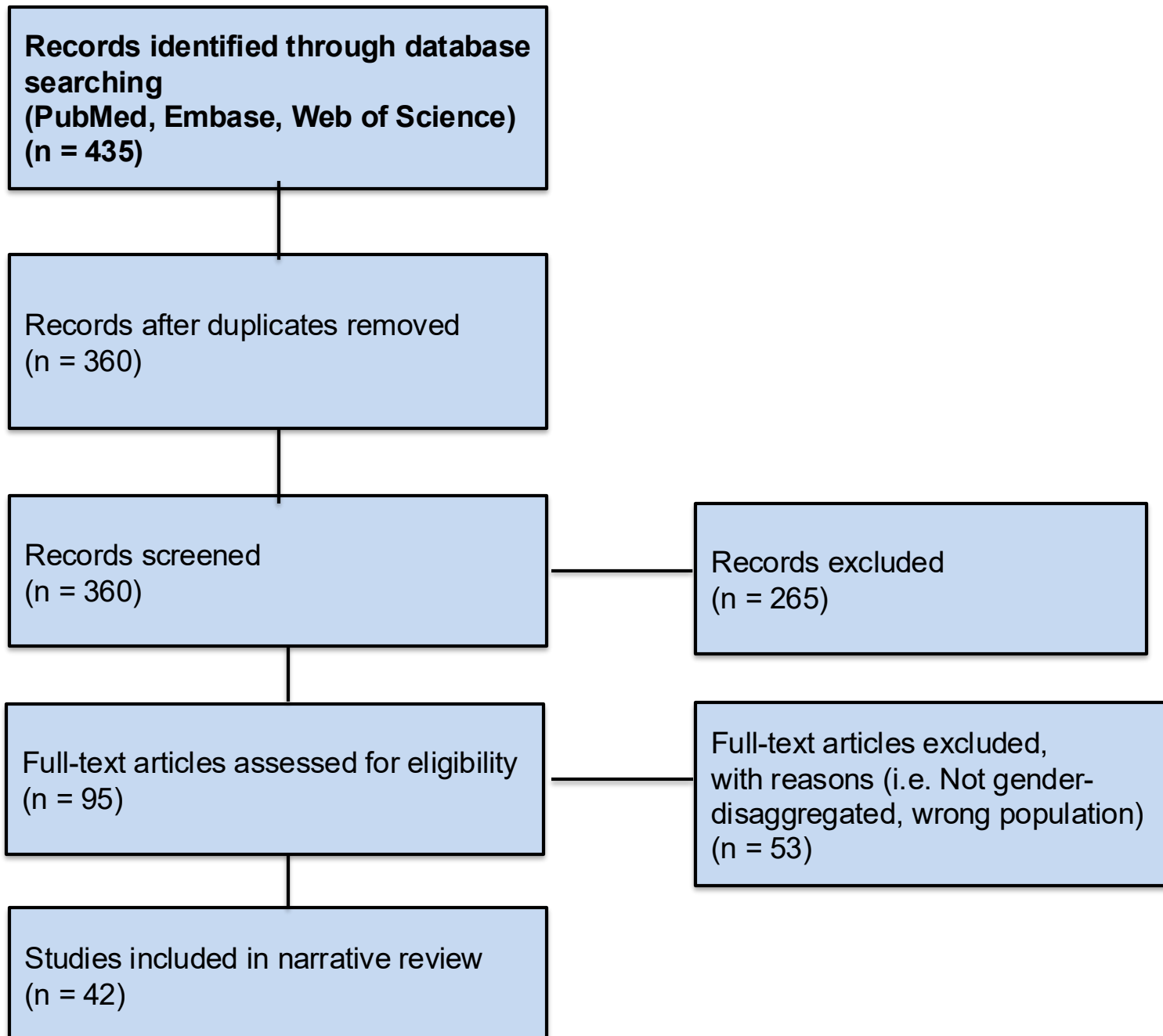

Supplement: Supplementary Figure 1 — Created in BioRender. Pinti, M. (2025) https://BioRender.com/1bnfxvg”. Footnote to supplemental material Flow chart. ”A formal systematic review was not feasible due to heterogeneity of study designs, outcomes, and lack of gender-disaggregated data; therefore, the evidence was synthesized narratively”. [file DataSheet1.pdf]
